# Instrumental Diagnosis of Placenta Accreta and Obstetric and Perinatal Outcomes: Literature Review and Observational Study

**DOI:** 10.37825/2239-9747.1060

**Published:** 2024-10-01

**Authors:** Maria A. Castaldi, Alessandro P. Torelli, Pasqualina Scala, Salvatore G. Castaldi, Antonio Mollo, Giorgia Perniola, Mario Polichetti

**Affiliations:** a Department of Medicine, Surgery, and Dentistry, University of Salerno, Baronissi, Italy; b High Risk Pregnancy Unit, University Hospital “San Giovanni di Dio e Ruggi d’Aragona”, Salerno, Italy; c Department of Gynecological, Obstetrical and Urological Sciences, “Sapienza” University of Rome, Rome, Italy

**Keywords:** Placenta accreta, Obstetrical outcome, Diagnosis, MRI, Ultrasound

## Abstract

**Aim:**

Placenta accreta (PA) is a condition where the placenta is pathologically adherent to the uterus due to a defect in the basal decidua with myometrium invasion by chorionic villi and is classified based on the depth of myometrial invasion by histology. However, ultrasound and magnetic resonance imaging have excellent accuracy. In this study, we investigated clinical benefits of early instrumental diagnosis of PA, especially in reducing maternal–fetal complications and improving perinatal outcomes. We also evaluated diagnostic accuracy of ultrasound and magnetic resonance imaging on placental invasiveness assessment.

**Methods:**

In this review and observational retrospective study, risk factors of PA were collected, and pregnant women underwent third-trimester ultrasound and magnetic resonance imaging (MRI) to evaluate the degree of infiltration. Imaging results compared to histological findings and surgical evaluation.

**Results:**

A total of 38 patients were diagnosed with at the University Hospital “San Giovanni di Dio and Ruggi d’Aragona”, Salerno, Italy, by second-trimester ultrasound with high sensitivity (100%) and accuracy (86%). Moreover, 37 of them performed MRI and 60.5% were diagnosed with Accreta, 7.9% increta, 10.5% percreta, and 21.1% not accrue with high sensitivity (100%), specificity (88.9%), and accuracy (97.4%). Histological assay confirmed MRI findings in 96.7% of cases. Risk factors of PA were age >35 years and previous CT scans. In unborn babies, mean 1-min Apgar was 4.3 (range, 3–6), and mean 5-min Apgar was 7.13 (range, 7–9).

**Conclusion:**

MRI could be a not-invasive, specific, sensitive, and accurate diagnostic tool for assessing the degree of infiltration in PA, and could guide clinical decisions, such as delivery plan, thus reducing perioperative and fetal complications.

## 1. Introduction

Placenta accreta spectrum (PAS), also termed abnormally invasive placenta (AIP), is a pathological obstetric-gynecological condition, caused by the absence of physiological placental detachment from uterine walls after childbirth, leading to massive bleeding, and being a life-threating condition for both pregnant woman and unborn baby. PAS is caused by a defect in the basal decidua, that invades the chorionic villi and trophoblast cells, and is classified based on the degree of adherence to the myometrium in three variants: Placenta Accreta (PA), where chorionic villi are attached to myometrium surface, in the absence of decidual layer, not invading muscle fibers; Placenta Increta (PI), where chorionic villi deeply invade the myometrium, without involving the serosa; and Placenta Percreta (PP), where villi reach the last uterine layer or the serosa, frequently involving near pelvic organs (especially the bladder) [1,2]. PAS can also be distinguished based on the type of anomalous placentation: abnormally adherent placenta with myometrium adherence without a real cleavage plane (PA), and abnormally invasive placentation (PI and PP), where manual and curettage removal are difficult, due to deep invasion by chorionic villi. Therefore, pre- and intra-partum diagnosis represent the crucially important step for the best therapeutic approach.

In 2019, the international society FIGO (International Foundation for Gynecology and Obstetrics) has introduced various grades of PA [1,3,4]. PA grade I, fitted shape: during manual delivery, secondment does not occur, and attempts of manual removal lead to profuse vaginal bleeding, that might require laparotomic surgery to directly evaluate the uterus and to perform a hysterectomy for histological evaluation. In this case, placenta cannot be distended without signs of invasion to the uterine walls. In PI grade II, invasive form, placenta appears as bluish or purple, with neovascularization spreading cranio-caudally towards the peritoneum without macroscopical placental invasion to the uterine wall. Conversely, chorionic villi are found within myometrial tissue and sometimes in the deep uterine vessels by histological evaluation. PP grade III is the most invasive form and is divided in: Grade 3a, where placental invasion is limited to the uterine serosa; Grade 3b, where placenta macroscopically invades only the bladder, and microscopically, chorionic villi are present within the uterine serosa and bladder wall; and Grade 3c, the highest invasion degree involving other tissues and pelvic organs [1,2,4,5,6].

Main risk factors are: placenta previa, with an increasing risk with age, from 2% in younger pregnant women (age <35 years in the absence of previous caesarean sections) to 39% (age >35 years with 2 or more previous caesarean sections); previous cesarean section, from 3% (no previous cesarean section) to 67% (>3 interventions); and maternal age >35 years [8,13,17,20]. Other risk factors are [12,23]: repeated curettage procedures; uterine scars or abnormalities (as in Asherman syndrome); ablations; previous uterine surgery, including myomectomy or radiofrequency thermal ablation; assisted insemination; endometriosis; and intra-uterine devices (IUDs), likely because they induce chronic inflammation, scars and tissue damage due to multiple insertions and removals; changes in uterine vasculature; and progesterone-induced endometrium thickening [16,19]. In the total absence of risk factors, incidence of PA is ~1 case in 22,000 births [23]. Recurrence is low (22**–**29% of cases).

PA is not associated with characteristics symptoms during pregnancy, except abnormal uterine bleeding in rare cases (metrorrhagia) and massive vaginal bleeding caused by incomplete placental detachment from myometrial wall, mostly during the third trimester. Usually, these episodes are not accompanied by pelvic pain, thus they represent life-threatening conditions, requiring emergency surgery. Other symptoms, although rare, can be abdominal pain, abnormal uterine activity, increased volume uterus, and bleedings during labor or pregnancy [24].

### 1.1. Pathogenesis

The most accredited pathogenetic hypothesis is a defect in the interface between endometrium and myometrium in scar areas produced by medical interventions, leading to abnormal decidualization, and anomalous and deep penetration of the extravillous trophoblast to trophoblast cell column and uterine connective tissue [14]. Epithelial cells loss their polarity, with cytoskeleton remodeling, cytokeratin replacement with vimentin, and acquisition of a migratory mesenchymal phenotype with infiltrating properties [25]. Decidua can invade muscular and serous layers of salpinges or of the abdominal cavity [25,26], and leads to uncontrolled invasion of the extra villous trophoblast throughout myometrium wall. These events are often triggered by abnormal scar formation and neovascularization after invasive procedures, such as manual labor during a previous pregnancy, or uterine curettage [18,19,25], and an unusual uteroplacental vascularization pattern is observed, with deeper arteries compared to normal pregnancies, and uterine vessel dilation [15]. Another etiopathogenetic mechanism is attributed to *in vitro* conception hormonal environment during implantation and placentation, because of a possible increased trophoblast invasion due to elevated serum estrogen levels, and abnormal trophoblastic growth caused by low serum estradiol levels and thin decidualized endometrium [34,35]. Only few studies have focused on molecular mechanisms associated with PAS, especially on inflammation and non-coding RNAs, including miRNAs, identified by next-generation sequencing of PAS-affected placentas [26–28].

### 1.2. Diagnosis

Prenatal diagnosis is especially important for improving maternal and new-born outcomes, by reducing morbidity and mortality associated with hemorrhagic complications [24]. In high-risk pregnancies, an accurate assessment of the uteroplacental interface using ultrasound is mandatory, and if the B-mode (grayscale) ultrasound does not provide a conclusive diagnosis, magnetic resonance imaging (MRI) or Doppler flowmetry can be considered useful complementary tools [32]. Although accuracy of prenatal diagnosis reaches 95% in centers with consolidated experience, PAS are undetected before birth in approximately half of cases in the entire population, and undiagnosed PA is suspected in cases of delayed placental expulsion within 30 min from delivery, failure to create a separation plan with attempts at manual secondment, and severe hemorrhages during placental traction [13].

#### 1.2.1. Ultrasound

Ultrasound is the first level exam for patients at risk or with suspected PAS [9,10,11], and various methods have been used to diagnose PA during second and third trimesters of pregnancy, also implementing standardization criteria [37,40], including loss of the “hypoechoic zone” under the placental bed, irregularities and attenuation of uterovesical interface, reduced thickness of retroplacental myometrium (<1 mm), detection of placental bulges, exophytic masses reaching the uterine serosa, placental gaps, and prominent vessels or lakes within placenta or myometrium.

Visualization of lacunae is the most useful criterion for PA diagnosis of placenta accreta in the second trimester of gestation, with a specificity of 78.6% [42]. Principal ultrasound signs of PAS are placental gaps o “Swiss cheese”, characterized by intraplacental lacunae or vascular spaces, that often appear linear-parallel in shape and extend from the placenta to the myometrium, exhibiting internal turbulent flow, also documented by abnormal patterns on color Doppler imaging. The presence of multiple lacunae (>6 lacunae with turbulent flow) is a sensitive marker of PP starting from week 15 of pregnancy [40]. Anterior myometrial thickening (<1 mm or not visible), measured between the echogenic uterine serosa and the free retroplacental space, is another ultrasound marker of PAS. Its reproducibility is low, especially in transvaginal ultrasound, as ultrasound measurement is operator dependent. Therefore, an integrated evaluation with other ultrasound signs and a comprehensive clinical evaluation are essential for accurate diagnosis and adequate management of suspected cases of PAS. Loss of retroplacental hypoechoic space due to damaged basal decidua allows a clear distinction between placenta and myometrium [43]. However, obliteration of retroplacental hypoechoic zone is present in both invasive and non-invasive conditions, thus this ultrasound sign lacks specificity with a high false positive rate (21%). Bladder space is the space between the uterus and the bladder, and is a continuous broad, thin, smooth hyperechoic line, while in PA this line is irregular and interrupted, due to increased vascularization [33]. In PP, this space could also appear with rupture, irregularity, thickening and increased vascularity, or swelling of the placenta into posterior bladder wall. This ultrasound sign has high sensitivity and specificity. Another ultrasound sign of suspicious PAS is the placental bulge, a deviation of the serosa from its normal plane caused by anomalous placental profusions towards adjacent organs [25].

Ultrasound should be carried out with full bladder (200**–**300 mL), while overfull bladder should be avoided because of vascular structure alterations and interface modifications, due to compression of the placental bed. In addition, the pitch angle, that is the border between placenta and myometrium, should be at 90°, while if it is close to 0°, it is more likely an artefact leading to possible false positives. Finally, probe pressure should be calibrated to correctly evaluate myometrial thickness. Ultrasound re-evaluation should also be performed at the second and third trimester of pregnancy, when ultrasound findings have greater specificity and sensitivity [41].

Although widely used, ultrasound is not as much specific for PAS in patients without risk factors, especially in those subjects without available clinical history (sensitivity of 62.8% and specificity of 53.5%, compared to a sensitivity of 90.7% and a specificity of 96.9% in patients with known clinical history) [41].

Use of 3D power Doppler ultrasound could improve the accuracy of prenatal diagnosis of PAS, providing more details on anomalous vascularization of the placenta, uterine serosa, and bladder wall, as a blood flow >15 cm/s is suggestive of accretism [37,38]. However, ultrasound signs alone cannot be used for PAS invasiveness classification, as there is no clear correlation with macroscopic aspects and histological findings. In FIGO guidelines [23], the use of grayscale ultrasound alone has been posed to have a sensitivity of 50**–**87%, increasing when power/color-doppler is associated (sensitivity of 90%). In 2016, the European Working Group on Abnormally Invasive Placenta (AIP) (now the International Society for PAS) has also proposed a standardized definition of ultrasound descriptors of PAS to improve comparability between studies, increase diagnostic accuracy and facilitate international collaborations [32], and this standardized terminology has now been included in the 2019 FIGO guidelines on prenatal diagnosis and screening of PAS [7].

#### 1.2.2. MRI

MRI is a second level exam for the proper diagnosis of PA, with high specificity and sensitivity after 24 weeks of gestations and can allow the assessment of posterior invasion and the depth of invasion of parametrium, myometrium, and bladder [44]. In MRI, myometrium and placenta can be clearly distinguished, as well as placental septa, normally regularly arranged [9]. In PAS, dark irregular intraplacental bands on T2-weighted images are observed [41]. Other imaging signs are heterogeneous signal intensity within the placenta and uterine bulging. In cases of PP, MRI is necessary for evaluation of bladder involvement [21]. Bladder profusion, an unfavorable placental position, or high maternal BMI are not contraindications for MRI [41,45].

### 1.3. Molecular markers

Different molecular markers have been developed by recent research to be used in the clinical setting. Approved PAS markers are: AFP (α-fetoprotein), highest between the 14 and 21 week of gestation; β-hCG (human chorionic gonadotropin), lower than normal values between the 11 and 12 week of gestation, while higher between the 14 and 21 week; and PAPP-A (pregnancy-associated plasma protein), highest between the 11 and 12 week [46].

Maternal serum alpha-fetoprotein (MS-AFP) is the major glycoprotein binder in fetal serum synthesized by the yolk sac in early pregnancy and later by fetal gastrointestinal tract and liver, and is present in the maternal serum because of active transport through the placenta or by diffusion across fetal membranes. MS-AFP is widely used as biomarker in the second trimester for prenatal screening, and high levels are present in PAS, and can pose indication in the second trimester to hysterectomy, especially in women with PP. Limitations of MS-AFP are: the small number of studies supporting the use of this marker; the lack of prognostic power during the first trimester for PAS and PAS-related complications; cut-off variability across studies; and the lack of specificity. Therefore, further research is needed to better evaluate diagnostic utility of MS-AFP also in differential diagnosis of PAS [47].

β-hCG is a glycoprotein produced by the trophoblast and the outer part of the embryo after conception and during pregnancy, and is composed by two subunits, alpha and beta, connected through non-covalent bonds. The alpha subunit is common to other gonadotropins, such as follicular stimulating factor, while the beta subunit is specific of β-hCG and is responsible for its biological functions. β-hCG production begins shortly after embryo implant in the uterus, usually around the sixth day after conception, and its levels rapidly increase to stimulate the corpus luteum to produce progesterone, essential for maintaining an endometrial layer suitable for embryo implantation and for supporting pregnancy in the early stages, before the placenta is fully functional [53]. This gonadotropin is also involved in other mechanisms, such as angiogenesis of uterine and placental blood vessels, placental development, and inhibition of maternal immunity against trophoblastic cells. Several studies have found that maternal serum β-hCG levels could be increased during PAS, already in the first trimester [52], and has a good specificity (68%) and sensitivity (53%) for PAS during the second trimester. Limitations of β-hCG are: great fluctuations during the first trimester of pregnancy, making it difficult to establish an accurate reference value for predicting PAS; and the lack of specificity for differential diagnosis between PAS and other conditions, such as spontaneous abortion, ectopic pregnancy and fetal anomalies.

PAPP-A is a glycoprotein produced by the syncitiotrophoblast during pregnancy, increasing until mid-gestation and then gradually decreasing until delivery. PAPP-A modulates uterine environment and promotes fetal growth through various mechanisms, including regulation of blood vessel formation at the maternal**–**fetal interface and modulation of growth factor activities [53]. PAPP-A levels can be decreased in PA, while other studies have shown an increase during first months of gestation and its levels are associated with blood volume loss. Limitations of PAPP-A are low specificity for differential diagnosis between PAS and physiological pregnancies, and the lack of studies investigating diagnostic accuracy of this test [54].

Cell-free Fetal DNA (cff-DNA) in maternal serum derives from syncytiotrophoblast and cytotrophoblast cells, that pass through the placenta to maternal bloodstream. In PAS, higher cff-DNA levels are associated with increased risk, likely because placental invasiveness causes a greater leak of fetal cells into maternal bloodstream [55]. Cff-DNA increases already at the end of the first trimester of pregnancy and is higher than those levels reported in normal pregnancies (10**–**18%). Therefore, cff-DNA fraction is proposed as an accurate biomarker of PAS, especially in suspicious cases with suggestive clinical or ultrasound findings [55].

Other markers have been analyzed to better understand the pathophysiology of PA. Indeed, different markers of inflammation have been involved in abnormal placentation such as systemic immune-inflammation index (SII) and other inflammatory. Platelet distribution width, mean platelet volume, neutrophil-to-lymphocyte ratio, and platelet-to-lymphocyte ratio, SII can be used to predict PAS in pregnant women with PP. The relationship between the histologic subtypes of PAS and inflammatory parameters should be investigated in more comprehensive studies [56].

Additionally, also abnormal maternal serum VEGF, TNF-alpha, IL-4, and IL-10 levels have been retrieved in the placenta accreta spectrum [57].

Finally, it has been hypothesized that NADPH oxidase (NOX), which since its discovery has been involved into a huge variety of inflammatory processes, can exert a possible role in abnormal implantation [58–60].

MicroRNAs (miRNAs) are small non-coding RNA molecules that play a crucial role in gene expression regulation. Specific miRNAs are overexpressed or downregulated during PAS compared to normal tissues, such as miR-210, miR-21, miR-141, and miR-155 [48,50,51]. In particular, miR-210 is involved in hypoxia responses and angiogenesis promotion, and their dysregulation is involved in PA development [28,29,30].

### 1.4. Management

Management of PAS requires a multidisciplinary team, including obstetricians, anesthetists, surgeons (experts in pelvic floor and urological surgery), hematologists, radiointerventionists, and neonatologists, especially in PP with involvement of organs and tissues. First, the team must define the degree of placental invasiveness for choosing the best surgical strategy, that can be conservative or radical, and delivery, usually planned as an elective caesarean section for stable patients between 34+0th and 35+6th weeks. For both approaches, the main goal is to avoid massive intra- and postoperative bleedings, resulting from angiogenesis and invasion of the chorionic villi. Therefore, placental removal should be performed, as residual placental material could further damage the myometrium, already compromised [36,39,61,62]. Because of increased risk of bleeding, blood products availability during surgery and post-operatively should be assessed before any invasive procedure [3,22,63–66]. Pre-term pregnancy termination should be considered only in case of severe obstetric complications such as, risk of preterm birth or heavily vaginal bleeding.

Conservative surgery aims to preserve as much uterine function as possible, while minimizing the risk of maternal complications, and is preferred in those women who want to maintain their future fertility or who want to avoid a hysterectomy. These strategies minimize the risk of bleeding and other post-operative complications, while simultaneously preserving reproductive capacity. However, this approach is not always feasible, and depends on PA extension and patient’s health status [64]. Conservative approaches are: selective placental removal of only those portions closely attached to the uterine wall, sparing normal placental tissue; manual separation techniques, with gentle manual separation from the uterine wall; use of hemostatic tampons or drugs to control bleeding while trying to remove the placenta; and uterine repair after placental removal that has caused an uterine injury.

Radical surgery is an extremely invasive and aggressive procedure and is of choice when the placenta is deeply infiltrated into the uterine wall, if other attempts of bleeding control have failed or are at high risk of complications, or there is a life-threatening condition for the mother. During this procedure, the uterus and all infiltrated uterine tissues are removed, to prevent serious bleedings and other complications. Hysterectomy can be total and is the most frequently performed surgery for PA, or subtotal, when PA does not involve the cervix. Radical hysterectomy is life-saving procedure when other methods of bleeding control are ineffective [60,67]; however, because of permanent loss of fertility, woman must be fully informed about risks and benefits of this procedure before radical hysterectomy, and shared decisions should be made together with medical team, considering her clinical situation, future pregnancy wishes, and long-term complications. Hysterectomy is associated with several complications, such as large blood loss, requiring intra- and post-operative blood transfusions.

Another approach for reducing bleeding is the use of hemostatic balloons or embolization of large uterine vessels [65]. Hemostatic balloons, also known as Foley balloons, are devices used to control bleeding during surgeries, and are inserted into the vaginal tract and inflated with saline solution to apply pressure on uterine walls, to compress and close blood vessels [63,66]. These balloons can be employed as part of a conservative surgical approach or as a temporary measure to control bleeding during surgery. These devices can be also inserted into the uterine cavity during placental removal attempts [49,64], as this approach can reduce bleeding and allow to safely perform surgery [64]. Uterine arterial embolization is a radiological procedure where small particles or embolizing agents are introduced into placental or uterine arteries, to interrupt blood flow and reduce the risk of excessive bleeding before and during surgery [65]. During embolization procedures, particles can pass through blood vessel walls and cause necrosis of surrounding tissues.

Segmental compression sutures are an alternative approach for bleeding control in occult retroperitoneal or colpo-uterine system hemorrhages [62].

## 2. Methods

### 2.1. Study design and population

In this study, we evaluated the impact of early instrumental PA diagnosis on clinical management, risk of maternal**–**fetal complications and perinatal outcomes. We also assessed diagnostic accuracy of ultrasound compared to MRI on placental invasiveness definition. A total of 38 patients were included in this observational study conducted at the High-risk Pregnancy Inpatients Unit, Obstetrics and Gynecology Department, University Hospital “San Giovanni di Dio and Ruggi d’Aragona”, Salerno, Italy. Patients were enrolled at the beginning of the second trimester and were divided in two groups based on risk of PAS (intermediate or high). PAS was diagnosed with ultrasound. Collected clinical history data were: previous CT scan or intrauterine surgery, including dilation, curettage, resectoscope polypectomy, myomectomy, or diagnostic hysteroscopy; BMI; use of IUDs; number of previous pregnancies; demographics; pre- and postoperative hemoglobin levels; the number of red blood cells units transfused; complications during hysterectomy; and other procedures performed. Subjects were divided in three groups based on PAS risk: low risk (no risk factors); intermediate risk (1**–**2 risk factors); and high risk (>3 risk factors). Patients were prepared with urethral stenting and catheters in hypogastric arteries, and four of them (with high suspicion of percretism) also underwent embolization of uterine arteries, to further limit massive bleedings. PAS diagnosis was made by histological examination performed on uteri derived from radical or partial hysterectomies; otherwise, PAS was proposed based on surgical evaluation during placental removal. Perinatal data were also collected and included 1-min and 5-min Apgar scores, intensive neonatal care requirements, use of intravenous catheters or invasive ventilation.

### 2.2. Ultrasound

First level ultrasound imaging was performed using a Voluson™ E8 ultrasound instrument equipped with RIC5-9 transvaginal and 9L high frequency 2D transabdominal probes. All patients were assessed with a suitable bladder volume (250**–**300 cc), and color Doppler and power Doppler settings were PRF 1.3 kHz for color Doppler and PRF 0.9 kHz for power Doppler, to better visualize placental status and minimize false positives and artifacts. Transabdominal investigation was firstly performed followed by transvaginal approach. Collected ultrasound findings were: presence of placental lacunae (Swiss cheese); irregularity or thinning of the hyperechoic uterine bladder**–**serous interface (bladder line); focal exophytic masses invading the bladder; reduced myometrium thickness (<1 mm); loss of the uteroplacental interface or of retroplacental hypoechoic space (clear space). Collected color Doppler ultrasound findings were: diffuse or focal turbulent flow within placental lacunae; hypervascularity of the bladder**–**serous interface; and dilated vessels beyond the peripheral subplacental zone. A re-evaluation ultrasound was performed at the beginning of the third trimester.

### 2.3. MRI

MRI without contrast was performed in all patients with suspicious ultrasound signs of PAS and intermediate-high risk and in patients with difficult ultrasound interpretation (e.g., obese or with posterior PP). MRI was carried out using a Philips Achieva D-stream 1.5T high field resonance, and a surface coil for body imaging was positioned on the anterior abdominal wall. Collected MRI findings included: uterine bulging (focal uterine swelling); heterogeneous placental signal intensity; presence of intraplacental dark bands on T2; and focal myometrial disruption. A score of 0**–**7 was then assigned based on the presence of MRI signs.

### 2.4. Statistical analysis

Data were analyzed using Prism (v.10.2.0; GraphPad software, La Jolla, CA, USA). Unpaired two-tailed t- or non-parametric Mann Whitney tests for two group comparison and Kruskal**–**Wallis test for three-group comparison were performed. Multiple linear regression was carried out for multivariate analysis. A P < 0.05 was considered statistically significant.

## 3. Results

### 3.1. Clinical characteristics and diagnosis of PA

A total of 38 patients were enrolled in this study and had a mean age of 36.6 years old (range, 25**–**45 years), and 66% of them aged >35 years. First ultrasound evaluation was performed in all of them during the second trimester of pregnancy, between week 16 and 22, and they all showed signs of PP or suspected accretism. Clinical characteristics are summarized in Table 1. Three patients (7.9%) had no risk factor for PAS, twelve (31.6%) 1**–**2 (intermediate risk), and 23 (60.5%) more than 3 factors (high risk). These latter two groups were further evaluated at the third trimester between week 27 and 30 by ultrasound and color Doppler ultrasound. At this second ultrasound, anomalous placental position was reported as central in 45.7% of cases (N = 16), anterior in 34.3% (N = 12), antero-lateral in 20% (N = 7), and posterior in 8.6% of cases (N = 3). MRI was then carried out in these patients except one for patient’s refusal, between week 27 and 36 (mean, week 34). PA was diagnosed by MRI in 67.4% of cases (N = 23), PI in 8.8% (N = 3), PP in 11.8% (N = 4), and no PAS in 23.5% of cases (N = 8). All 38 patients were managed with elective cesarean delivery, and 8 of them (21%) with conservative surgery, while 30 (79%) with radical hysterectomy. Histological examination was carried out on the 30 radical hysterectomy specimens and 23 cases were confirmed as PA, 5 with PI, and 2 with PP. Patients aged >35 years (66.7%), with previous CT scans (68.5%), and with at least one previous invasive intrauterine procedure or myomectomy (72.4%) had a higher prevalence of PAS compared to other subjects.

### 3.2. Ultrasound in PAS diagnosis

In our cohort, ultrasound findings in the first trimester were not specific, while diagnostic accuracy increased during second and third trimesters, especially when associated with color Doppler ultrasound for evaluation of hypervascularization in non-physiological areas. In the entire group, 3 women (7.9%) had PP without ultrasound signs of infiltration, while the remaining 92.1% of subjects showed at least one ultrasound sign of PA. Therefore, ultrasound displayed high sensitivity (100%) and very low specificity (38%), with a positive predictive value (PPV) of 85% and negative predictive value (NPV) of 100%, and a good accuracy (86%). However, ultrasound specificity significantly decreased in patients without risk factors, posterior PP, and unclear ultrasound images (57.4**–**85.7%). Moreover, no recurrent ultrasound signs were observed among patients.

### 3.3. MRI findings of PA

In our study, 37 out of 38 patients performed an MRI, confirming a grade I–III infiltration in 81% (N = 30) of subjects. In details, PA or PI was suggested in 67.6% of cases (N = 25), PP in 10.8% (N = 4), and no PAS in 21.6% of subjects (N = 8). MRI findings were compared with histological examinations, and MRI-driven diagnosis was confirmed in all cases, except one where PP was made instead of PI. Therefore, MRI displayed extremely high sensitivity (100%) and specificity (88.9%), as well as high PPV (96.7%), NPV (100%), and accuracy (97.4%).

### 3.4. Clinical outcomes

In our study, all pregnant women were managed with elective caesarean section between week 32 and 37 of gestation (mean, week 35.2), and 78.9% of them underwent hysterectomy. Intraoperative blood losses were managed with blood flow blocking procedures, ureteral stents, and catheters in hypogastric arteries, while embolization was performed in only 4 subjects with PP. Mean preoperative hemoglobin levels were 10.7 g/dL (range, 9.0**–**12.2 g/dL), and they significantly decreased after surgery (mean, 8.7 g/dL; range, 7.1**–**10.1 g/dL) with a mean loss of 2.0 g/dL per patient (range, 0.3**–**3.9 g/dL), requiring at least one packed red blood cells (maximum 7) transfusion (Table 2). Finally, all 38 newborns were admitted to the neonatal intensive care unit and received umbilical vein cannulation and invasive ventilation. Regardless of the degree of placental invasiveness, newborns displayed a mean 1-min Apgar score of 4.3 (range, 3**–**6) and 5-min Apgar of 7.1 (range, 7**–**9), suggesting that perinatal outcomes were more related with prematurity rather than PAS.

## 4. Discussion

PAS is a potential life-threatening condition for both the mother and the fetus, due to high risk of massive bleedings and blood loss. Therefore, an accurate and early diagnostic definition, especially for the degree of placental invasiveness to uterine and near tissues and organs, is extremely important to better define treatment strategies in these patients. In our observational study, we investigated diagnostic power of ultrasound and MRI in definition of PAS, showing the superiority of MRI in diagnostic definition, allowing a better clinical management also reducing obstetric and neonatal complications [60–68].

Ultrasound is a first level instrumental test for screening for PAS, and for initial identification of main risk factors, including PP and positioning, isthmocele, pregnancy on scars, and uterine myomas or anomalies [69–72]. Ultrasound is not indicative in patients with limited anamnestic information, as is important to first formulate a PAS suspect and to risk-stratify patients (low, intermediate, and high). Indeed, collection of anamnestic data significantly improved ultrasound diagnostic power in our study, increasing its specificity from 62.2% to 95.8%. Ultrasound signs of PAS considered in our investigation were: placental gaps, bladder line, exophytic focal masses, reduced myometrial thickness, loss of uteroplacental interface and of retro-placental hypoechoic space. However, these signs, although indicative, are not pathognomonic of PA, indirectly identify signs of accreta, and can not accurately assessed the depth of invasion. Indeed, also in our study, specificity was very low (38%), with high false positive cases [31–34]. Conversely, association with color Doppler ultrasound can increase diagnostic accuracy, allowing the study of anomalous placental neovascularization [34,38].

MRI identifies local signs of invasion, while its ability to determine the precise extent of invasion is limited. In our study, MRI showed excellent sensitivity (100%) and specificity (88.88%), particularly for patients with posterior placenta previa or obesity, where ultrasound was less sensitive. Different from other published studies, we showed a higher specificity of MRI in diagnostic PAS definition compared to ultrasound. Despite being an expensive diagnostic tool, MRI significantly reduces the number of false positives, avoiding unnecessary invasive procedures and allowing personalized approaches for childbirth and PAS treatment. Although both ultrasound and MRI provided useful information for PAS diagnosis and risk stratification, only histopathological examination or intraoperative evaluation confirmed or not imaging findings, thus remaining the gold standard for PAS diagnosis [40,45,46].

Treatment strategies for PAS patients should be always planned in advance, trying to minimize emergency cases occurrence. Therefore, elective cesarean sections should be preferred, even in those subjects at low risk of accreta, as PP could be present. Elective surgeries can also minimize the bleeding risk during surgery; however, despite the presence of a multidisciplinary team, including a radiologist for arterial embolization, intra- and postoperative transfusions were performed in the majority of our patients, confirming that PA is a life-threatening condition if not well managed.

Ultrasound and MRI are crucial tools for PA management, together with collection of anamnestic data. However, ultrasound shows low diagnostic sensitivity and specificity in patients without risk factors of PAS or without clinical anamnestic data. Therefore, MRI should be preferred in these subjects, as it offers significant advantages in visualizing placental infiltration, reducing the number of false positives, and improving treatment planning. Even so, histopathological examination remains the gold standard for diagnosis, and elective birth planning is essential to minimize risks and improve mothers’ and newborns’ outcomes [24,70].

Despite a precise risk stratification before delivery by ultrasound and/or MRI, conservative procedures are still unsafe with higher incidence of massive bleedings during surgery and in the post-operative period. Moreover, post-hysterotomy scar in conversative approaches could represent a very high-risk area for placental insertion in subsequent pregnancies. In conclusions, our results support the importance of early diagnosis and risk stratification for a better and personalized clinical management with elective caesarean section and appropriate hemostatic approaches.

## Figures and Tables

**Table 1:** Clinical characteristics of the patients included in the present study.

Characteristics	Cohort N = 38
Mean age, years (range)	36.6 (25**–**45)
Age >35 years old, %	66.7%
Mean number of pregnancies, n (range)	2.3 (1**–**4)
Previous pregnancies, n (%)	
0	4 (10)
1	3 (7)
2	17 (45)
≥3	14 (38)
Mean gestational age, weeks (range)	35.2 (32**–**37)
Mean BMI, Kg/m^2^ (range)	23.55 (15**–**30)
Underweight, n (%)	3 (7)
Overweight, n (%)	13 (33)
Obese, n (%)	1 (3)
Previous surgery, median (range)	2 (0**–**4)
Cesarean section, n (%)	26 (68.5)
Previous intrauterine procedures	13 (33.3)
Previous myomectomy	4 (9.3)

**Table 2:** Clinical outcomes.

Characteristics	Mean (range)
Pre-operative hemoglobin, g/dL	10.7 (9**–**12)
Post-operative hemoglobin, g/dL	8.7 (7.1**–**10.1)
Blood loss, g/dL	2.0 (0.3**–**3.9)
Red blood cell transfusion, n	2.8 (2**–**7)
Fetal 1-min Apgar	4.3 (3**–**6)
Fetal 5-min Apgar	7.4 (7**–**9)

## Data Availability

Data are available upon request by the corresponding author.
